# Retraction: Metformin-enhanced cardiac AMP-activated protein kinase/atrogin-1 pathways inhibit charged multivesicular body protein 2B accumulation in ischemia–reperfusion injury

**DOI:** 10.3389/fcell.2026.1794645

**Published:** 2026-02-02

**Authors:** Frontiers Editorial Office

The journal retracts the 5 February 2021 article cited above.

Following publication, concerns were raised regarding the integrity of the data due to an overlap between figure 2A and figure 4A. The authors failed to provide a satisfactory explanation during the investigation, which was conducted in accordance with Frontiers’ policies. As a result, the data and conclusions of the article have been deemed unreliable and the article has been retracted.

This retraction was approved by Chief Editors of Frontiers in Cell Development and Biology and the Chief Executive Editor of Frontiers. The authors do not agree with this retraction.

